# A LASSO-based approach to analyzing rare variants in genetic association studies

**DOI:** 10.1186/1753-6561-5-S9-S100

**Published:** 2011-11-29

**Authors:** Jennifer S Brennan, Yunxiao He, Rose Calixte, Epiphanie Nyirabahizi, Yuan Jiang, Heping Zhang

**Affiliations:** 1 Department of Epidemiology and Public Health, Yale University, New Haven, CT 06520, USA

## Abstract

Genetic markers with rare variants are spread out in the genome, making it necessary and difficult to consider them in genetic association studies. Consequently, wisely combining rare variants into “composite” markers may facilitate meaningful analyses. In this paper, we propose a novel approach of analyzing rare variant data by incorporating the least absolute shrinkage and selection operator technique. We applied this method to the Genetic Analysis Workshop 17 data, and our results suggest that this new approach is promising. In addition, we took advantage of having 200 phenotype replications and assessed the performance of our approach by means of repeated classification tree analyses. Our method and analyses were performed without knowledge of the underlying simulating model. Our method identified 38 markers (in 65 genes) that are significantly associated with the phenotype Affected and correctly identified two causal genes, *SIRT1* and *PDGFD*.

## Background

Although genome-wide association studies based on the common disease/common variant assumption have identified many disease-causing genetic variants, the variants usually explain only a small percentage of disease risk [1]. This suggests that rare variants are potentially important to the unexplained risk. Common variant association methods have been extensively developed, but because the frequency of a rare variant is so low (less than 1%) it seems imprudent to apply routine statistical procedures to analyze a low minor allele frequency (MAF). Even in large-scale genome-wide association studies, the rare variant at a single marker appears so infrequently that typical statistical methods are invalid or inapplicable.

Given the discrepancy between relatively high common disease prevalence and low MAF of a rare variant single-nucleotide polymorphism (SNP), it is biologically unlikely that the bulk of disease risk is attributable to a single rare variant. Instead, it is more likely that multiple rare variants increase disease risk [2]. Accordingly, although only one rare variant may be expected to occur in a given individual, any occurrence from a particular group of rare variants would explain a relatively large percentage of disease risk. Although not ideal, it seems reasonable, and perhaps inevitable, to appropriately group rare variants into artificially made markers to test the association of the group with a specific phenotype.

Several methods for exploring associations between multiple rare variants and disease risk have been proposed [3-6]. A primary goal in each of the current methods is to construct a set of candidate markers from the original set of SNPs by collapsing rare variants over a predefined functional grouping unit, such as a gene or nearby genomic region. These candidate markers are then considered in an association analysis. For example, Morris and Zeggini [6] suggested collapsing by using either an indicator variable describing the presence of rare variants or a quantitative variable for the proportion of the variants that carry at least one copy of the minor allele. Li and Leal [3] proposed using the combined multivariate and collapsing (CMC) method. Their approach divides markers into prespecified subgroups, such as genes, and then collapses the genotypes within each group. These approaches are reasonable, but it is useful to explore additional methods. In the data from Genetic Analysis Workshop 17 (GAW17), for example, there are so few SNPs in a particular gene that collapsing across genes alone does not sufficiently increase the MAF of a created marker.

We present a method that attempts to remedy some of the current problems with two chief improvements: First, SNPs are combined only when they show a joint effect on the response; and, second, SNPs that are not in a gene are grouped according to their positions on a chromosome. We applied this method to the GAW17 data, and our preliminary results suggest that this approach is promising. Our method and analyses were performed without knowledge of the underlying simulating model.

## Methods

### Data

Our analysis focuses on the GAW17 case-control data (with outcome phenotype Affected), which consists of 697 unrelated individuals from 17 different population (race or ethnicity) groups. Four other risk factors are included in the data: race or ethnicity, age, sex, and smoking status. There are 200 phenotype replicates based on the same genotype information. Among the 24,487 SNPs in the data, 87.2% (*n* = 21,355) of them have MAF < 0.05, 74.0% (*n* = 18,131) of them have MAF < 0.01, and 38.5% (*n* = 9,433) of them have MAF < 0.001. See the GAW17 simulation materials for additional details on the data [7].

### Preprocessing

Because we analyzed the data without knowledge of the underlying simulation process, we undertook two preprocessing steps: assessing Hardy-Weinberg equilibrium (HWE) and evaluating population structure by means of stratification analysis. These steps were taken as a necessary precaution using PLINK [8].

HWE was evaluated to determine whether the genotype frequencies obtained in the data match those expected under HWE conditions. Consequently, we excluded from further analyses all SNPs with MAF > 0.05 and all SNPs that failed the HWE test at *p* < 0.0001 [9]. We also examined potential population stratification and concluded that it is appropriate to consider race or ethnicity in our association analysis.

### Combining SNPs into new markers using the LASSO technique

The crux of our method is to produce composite markers using the least absolute shrinkage and selection operator (LASSO) procedure, which represents SNPs in a predefined group. This is a two-step process: First, given a sufficiently large number of rare variant SNPs within a gene, we group rare variant SNPs within their gene. Then all remaining SNPs that are of limited number in any given gene are grouped according to their positions on a chromosome. Furthermore, SNPs are combined only when they show a joint effect on the response using the LASSO method [10].

Specifically, we first run the LASSO procedure on SNPs within the same gene and then again on the sparse SNPs within a group that spreads over different genes. A 0–1 dummy variable is created for each SNP based on the presence or absence of the rare variant. Then, linear combinations of the selected dummy variables are considered by using the LASSO procedure. Even though most of the dummy variables are 0, their linear combination is far more likely to be nonzero. In fact, we include only those linear combinations that are nonzero in at least 5% of the subjects. This ensures that the new markers are not rare.

Covariates can be readily incorporated into the LASSO technique by treating them as additional predictive variables. If particular covariates are known to be significantly associated with the phenotype, then they will not be penalized when running the LASSO procedure. Otherwise, the covariates are also penalized and subject to selection. In our analysis of the GAW17 data, the covariates (race or ethnicity, age, sex, and smoking) were not penalized. The Akaike information criterion (AIC) was used to select the best LASSO tuning parameter (the penalizing coefficient) corresponding to the best-fitting model. The entire LASSO path was computed using the R package glmnet [11].

## Results and discussion

The GAW17 data consist of 200 phenotype replications based on the same genotype information; this affords us the ability to conduct our marker selection method on one of the replicates and to use the remaining 199 replications to evaluate the performance of our method. We generated new markers on the first phenotype replication according to the SNP screening process, as introduced in the Methods section.

To take advantage of the remaining 199 phenotype replications based on the same genotype information, we assessed our new markers using an association analysis with the phenotype Affected. Any appropriate association method could have been used, although we opted to evaluate performance by conducting classification tree analyses [12] on the remaining 199 replications.

The classification analysis considered all acceptable markers produced by our method and adjusted for sex, age, smoking status, and race or ethnicity covariates. Thus for each replicate we constructed a classification tree using the same 493 markers and the phenotype Affected; 199 classification trees were constructed in total. We used the R package rpart for this analysis and applied the default setting of the rpart function. We took advantage of all the simulated replicates to evaluate the effectiveness of our method. In practice, our method can be used with or without replicates. When there are no additional phenotype replications, markers can be constructed and then used to build a tree. To evaluate the significance of the identified markers, one can generate additional data under the null hypothesis by randomly permuting affection status. This has been shown to be an effective technique in previous studies [13]. In the present case, however, further investigation is necessary to evaluate issues related to possible overfitting.

Heuristically, more frequently appearing markers across all of the 199 association analyses have a higher chance of contributing to the phenotype. For each of the 199 constructed association analysis trees, the null hypothesis is that the outcome phenotype, Affected, is independent of the markers, conditional on the covariates. Our screening process, as described in the Methods section, produced 493 nonrare linear combinations of the dummy variables (made from SNPs). Thus under the null hypothesis of no association (independence) between phenotype and any marker, each of the 493 generated markers has an equal chance of being selected to split a node in a tree. The probability that a marker will be selected at any particular split is 1/493. In addition, under the null hypothesis of independence, whether the marker is selected as a splitting variable in a previous split does not effectively change the probability that the same marker will be selected as a splitting variable in subsequent splits.

Denote the size (number of splits) of the *i*th tree for *i* = (1, 2, …, 199) as *s_i_*, and denote the total number of splits selected by all 199 trees as . Therefore under the null hypothesis of no association, the number of times that a specific split occurs across all 199 trees follows a binomial distribution Bin(*S*, 1/493)*.* Although there are several ways to adjust for the false-positive rate resulting from multiplicity, we used the Bonferroni correction to err on the side of caution. Thus using a Bonferroni-corrected significance level of 0.05, we find that the genome-wide-significance level is 0.05/493. Therefore a particular split is considered genome-wide significant if it meets or exceeds the quantile value of 0.05/493 under Bin(*S*, 1/493); therefore 14 occurrences across the 199 analyses is the critical value.

Our method identified 38 newly generated markers (composed of 65 genes) that are significantly associated with the phenotype Affected. The mean number of markers used per tree was 11.37 with a standard deviation of 3.98. In Table 1, we report the 18 new markers that appeared most frequently across each of the 199 trees. Aside from the sixth most significant marker, which is composed of multiple genes, all of the other 17 markers are composed of only one gene. Two causal genes, *SIRT1* and *PDGFD*, are correctly identified [7]. Both genes include multiple SNPs whose coefficients in the simulation model are relatively large (known after the simulation model was revealed).

**Table 1:** Top identified signals: frequency table of tree regression using LASSO markers, *n* = 199

Frequency	Chromosome	Gene
55	1	*LAMB3*
49	5	*PCLKC*
40	6	*MDN1*
34	4	*PDLIM5*
31	1	*KIF17*
28	10	*SIRT1*,* *HERC4*, *MYPN*, *PBLD*, *CXXC6*, *CCAR1*, *VPS26A*, *HK1*, *C10ORF35*, *H2AFY2*, *AIFM2*, *LRRC20*, *EIF4EBP2*, *NODAL*, *KIAA1274*, *PRF1*, *ADAMTS14*, *PCBD1*, *SLC29A3*, *CDH23*, *CAMK2G*
28	18	*TXNDC2*
28	20	*CYP24A1*
26	18	*EMILIN2*
26	1	*ARHGEF10L*
23	17	*ARHGEF15*
23	3	*GOLGB1*
22	1	*KIAA0133*
19	2	*LY75*
19	7	*RELN*
18	11	PDGFD*
18	21	*BRWD1*
18	6	*POU5F1*

Asterisks indicate correctly identified markers.

## Conclusions

We propose a novel approach to analyzing rare variant data by incorporating LASSO variable selection. This is a relatively easy-to-implement approach. The results from analyzing the GAW17 data suggest that the new approach can be useful for analyzing rare variant data.

We made use of all phenotype replicates provided by GAW17; the first replicate was used to generate markers, and the remaining replicates were used to evaluate the performance of the proposed method. In a real data analysis, however, the goal is typically to apply, not evaluate, the method. Chen et al. [13] presented a technique to evaluate significance that has proven to be successful. Their method centers on creating additional data sets that are generated under the null hypothesis by randomly permuting affection status. Additional investigation is necessary to assess possible overfitting.

Our method and analyses were performed without knowledge of the underlying simulating model. It should be noted, however, that the false-positive rate from the GAW17 data analysis was high because of the low level of signal, in particular, the low MAF of the rare variant SNPs.

## Competing interests

The authors declare that there is/are no competing interests.

## Authors’ contributions

EN, JSB, RC, YH, and YJ carried out the analyses. JSB and YH drafted the manuscript. HZ conceived and led the analysis, and revised and finalized the manuscript. All authors read and approved the final manuscript.
